# Application of VR360 for Clinical Reasoning Development in Occupational Therapy Students in Handling Complex Behavioural Symptoms in Individuals With Dementia

**DOI:** 10.1155/oti/7492246

**Published:** 2026-05-19

**Authors:** Frank Ho-yin Lai, Ben Chi-bun Yip, Eddie Yip-kuen Hai, David Wai-kwong Man

**Affiliations:** ^1^ School of Communities and Education, Faculty of Health and Wellbeing, The Northumbria University, UK, northumbria.ac.uk; ^2^ School of Medical and Health Sciences, Tung Wah College, Kowloon, Hong Kong, twc.edu.hk; ^3^ Mental Health Research Centre, The Hong Kong Polytechnic University, Kowloon, Hong Kong, polyu.edu.hk; ^4^ Faculty of Health Sciences and Wellbeing, University of Sunderland, Sunderland, UK, sunderland.ac.uk

## Abstract

Managing behavioural and psychological symptoms of dementia (BPSD) demands advanced clinical reasoning, yet traditional teaching often falls short. Virtual Reality 360° (VR360) technology offers immersive learning that may strengthen competence in dementia care. This study explored whether occupational therapy (OT) students’ reasoning improved after a VR360 module addressing BPSD such as hoarding, wandering, low mood and daily living challenges. Sixty students (35 female, 25 male; undergraduate and postgraduate) participated. Using a mixed‐methods, single‐group pre–post design, students completed the Clinical Reasoning Assessment Tool before and after four VR360 scenarios. Large improvements emerged across domains: content (*d* = 1.94), procedural (*d* = 2.11) and conceptual (*d* = 2.08). Qualitative themes highlighted confidence, cue recognition, empathy and personalised care. Findings suggest VR360 is feasible and effective for OT education.

## 1. Introduction

Dementia is a progressive neurological disorder that significantly impacts cognitive function, daily living activities and behaviour, affecting millions globally [1]. Dementia is a complex syndrome characterised by a progressive decline in cognitive functions, including memory, language, perception and executive functioning, which significantly interfere with daily living skills [2]. Behavioural and psychological symptoms of dementia, also known as BPSD, include emotional disturbances like depression and anxiety, as well as behavioural issues such as aggression and wandering. These symptoms can significantly impact the quality of life and complicate care management [3]. Occupational therapy (OT) plays a crucial role in managing these symptoms by focusing on non‐pharmacological interventions that are person‐centred and context‐specific [4]. Clinical reasoning in OT is a dynamic and iterative process that involves gathering information, analysing data and making decisions to guide intervention strategies [4]. It is a client‐centred approach that considers the individual’s needs, preferences and environmental factors [5]. In the context of dementia care, clinical reasoning is particularly important due to the heterogeneity of the condition and the complexity of BPSD [6, 7]. Occupational therapists use various frameworks and models to guide their clinical reasoning. For example, the person–environment–occupation model emphasises the interplay between the individual, their environment and the activities they engage in. This model is particularly useful in dementia care, as it allows therapists to identify environmental triggers for BPSD and tailor interventions to enhance participation in meaningful activities [7].

Virtual reality (VR) technology, particularly 360° (VR360) videos, has emerged as a powerful tool in education and training across various healthcare professions, including OT and allied health fields [8]. This immersive technology offers unique opportunities for skill acquisition, clinical simulations and patient‐centred care. One of the most significant applications of VR360 is in creating realistic clinical simulations for training healthcare professionals [9]. These simulations provide a safe and controlled environment for students to practise and master skills without risking patient harm. For example, a study involving nursing, social education and OT students demonstrated that VR simulations using 360° videos prepared students for complex clinical situations, such as patient assessments and communication scenarios [10]. Additionally, the use of realistic scenarios in VR simulations has been shown to enhance students’ emotional engagement and learning outcomes. Students reported that the realism of the videos was critical for their learning experience, as it allowed them to reflect on clinically relevant situations and achieve a deeper understanding [9, 11].

With the support of a research and development fund, the VR360 learning module named ‘Managing Behavioural Challenges in Dementia Care: Occupational Therapist Perspective’ was designed with four interactive scenarios that simulate common behavioural issues encountered in dementia care.

Despite growing interest in VR‐based education across health disciplines, there remains a notable gap in empirical evidence specifically examining VR360’s impact on OT students’ clinical reasoning in the context of BPSD management in dementia care. Existing studies have largely focused on general healthcare simulation rather than the specialised reasoning demands of OT practice with dementia populations. Furthermore, little is known about how individual learner characteristics—such as gender, education level and prior clinical experience—moderate the effectiveness of VR360 interventions. This study addresses that gap by providing preliminary evidence of the feasibility and potential effectiveness of a purpose‐built VR360 module targeting OT students’ clinical reasoning across four BPSD scenarios.

### 1.1. Research Objectives

This study aimed to explore whether OT students’ clinical reasoning improved following engagement with a four‐scenario VR360‐based learning module, providing preliminary evidence of feasibility and potential effectiveness. Additionally, the study aimed to explore how immersive simulation experiences influence students’ ability to identify, interpret and respond to behaviours such as hoarding, wandering and disorientation, low mood and suicidal ideation and challenges with daily living tasks. Additionally, the study aimed to assess whether students’ gender and pre‐exposure to clinical placement experience contribute to differences.

## 2. Methods

### 2.1. Research Design

This study will adopt a mixed‐methods, quasi‐experimental, single‐group pre–post design to evaluate the impact of immersive VR (VR360) learning on clinical reasoning in dementia care. Both quantitative and qualitative data will be collected to assess changes in clinical reasoning and explore students’ learning experience. A randomised controlled trial (RCT) was considered but deemed impractical and ethically challenging in this context. All participants were enrolled in the same module, and withholding access to the VR360 intervention could have introduced inequities in learning opportunities. Additionally, resource constraints (limited VR equipment and technical support) precluded simultaneous delivery to separate randomised groups. Therefore, this design was selected to provide preliminary evidence of feasibility and potential effectiveness, informing future controlled trials.

### 2.2. Participants

We planned to recruit approximately 60 OT students based on the practical consideration of the cohort size enrolled in the relevant modules during the study period and to ensure adequate statistical power for the study objectives. Based on anticipated large pre–post changes in clinical reasoning [12] and *α* = 0.05, this sample size provides power > 0.80 for detecting within‐subject improvements using paired analyses. This target was chosen to ensure feasibility and allow subgroup MANCOVA analyses by gender, education level and prior dementia care experience. Inclusion criteria include current enrolment in an OT programme, participation in a relevant module, the ability to provide informed consent, proficiency in English and willingness to engage in VR‐based learning and reflection activities. Students would be excluded if they have extensive prior experience in dementia care (e.g., as a relative or through more than 6 months of full‐time work as a formal caregiver), known sensitivity to VR environments (such as motion sickness or epilepsy), inability to participate in English‐language activities or lack of access to necessary technology if remote participation is offered.

### 2.3. Instrumentation

The Clinical Reasoning Assessment Tool (CRAT) [13] was used to assess students’ self‐reported clinical reasoning skills before and after the VR360 intervention. The CRAT comprises three subscales, content knowledge, procedural knowledge and conceptual reasoning, each scored on a 10‐point visual analogue scale (VAS) with four descriptive anchor levels: beginner (0–2.5), intermediate (2.5–5.0), competent (5.0–7.5) and proficient (7.5–10.0). Content knowledge addresses the foundational understanding a student brings to a case, including biological and physical sciences and the International Classification of Functioning, Disability and Health (ICF) framework, focusing on identification of facts rather than their interpretation. Procedural knowledge captures the application of appropriate examination techniques and psychomotor skills, encompassing what skills to perform, when to perform them and how to perform them effectively. Conceptual reasoning assesses the cognitive and metacognitive integration of information—the interrelationship and synthesis of data upon which clinical judgement is made, utilising reflection and self‐awareness.

Regarding psychometric properties, the CRAT has demonstrated face validity in physical therapy (PT) and OT educational contexts [14]. Longitudinal construct validity evidence has been reported supporting its utility in tracking procedural and conceptual reasoning development across a PT curriculum [15]. Concurrent validity is partially supported by Riopel et al. [14], who found strong and statistically significant student–faculty correlations for procedural knowledge in both PT (*r* = 0.679, *p* = 0.002) and OT (*r* = 0.676, *p* = 0.003) students and for conceptual reasoning in OT students (*r* = 0.505, *p* = 0.039). Formal reliability data for the CRAT have not yet been established in the published literature [14]; however, this limitation is consistent with the broader challenge of developing credible measures for clinical reasoning assessment across the health professions [16]. The CRAT’s use in this study aligns with its intended purpose as a structured self‐reflection tool to facilitate and assess clinical reasoning development, rather than as a psychometric outcome measure in the traditional sense.

### 2.4. VR360 Learning Module and Scenarios

This VR360 module presents four immersive scenarios that simulate common behavioural challenges encountered in dementia care. Each scenario is designed to help OT students observe, assess and respond to complex behaviours whilst applying their clinical reasoning. The development of this VR360 module emphasises experiential learning and reflective practice, which aligns with Kolb’s model by providing concrete experiences through immersive simulations. Learners can actively experiment and reflect on their experiences, facilitating deeper learning and understanding [17, 18]. Additionally, VR360 supports Schön’s reflective practitioner model by enabling learners to reflect on their actions in a simulated environment. This reflection‐in‐action helps develop professional skills and decision‐making abilities [18].

#### 2.4.1. Scenario 1: Hoarding Behaviour

In this scenario, students are placed in a 360° view of a client’s living room and kitchen within a residential care facility. The client, an elderly woman with moderate dementia, is observed hiding garments and small household objects in drawers, under cushions and in her bedroom. The environment is visibly cluttered, presenting potential safety hazards. Students are expected to identify the cognitive and environmental factors contributing to the hoarding behaviour, assess the associated risks and propose appropriate strategies such as environmental modifications and client‐centred interventions to manage the behaviour safely and respectfully.

#### 2.4.2. Scenario 2: Wandering and Disorientation

In this scenario, students are placed in a 360° view of a residential care facility, including a hallway, a common area and the surrounding community. The client, with moderate‐stage Alzheimer’s disease, is observed walking purposefully through the facility, expressing a desire to ‘go home before the children return from school’. He appears confused about his location and becomes increasingly distressed when redirected.

The environment includes subtle cues such as signage, familiar objects and the presence of passers‐by, allowing students to assess how these elements influence the client’s behaviour. Students are expected to identify signs of cognitive disorientation and wandering, assess the safety risks associated with elopement and apply person‐centred communication strategies to de‐escalate the situation. They are also encouraged to evaluate cognitive, environmental and emotional factors and to propose appropriate interventions such as environmental modifications, reminiscence‐based redirection and strategies to support the client’s well‐being.

#### 2.4.3. Scenario 3: Low Mood and Suicidal Ideation

In this scenario, students are placed in a 360° view of a private bedroom and an adjacent lounge area within a residential care facility. The client, a 74‐year‐old woman with early‐stage vascular dementia, is observed sitting alone in her room with the curtains drawn and minimal engagement in her surroundings. She speaks in a low tone, expressing feelings of hopelessness and making statements of despair. The environment is quiet and dimly lit, with personal items such as family photos and books visible but untouched. Students are expected to recognise signs of low mood and potential suicidal ideation, assess the emotional and cognitive factors contributing to her mental state and apply appropriate communication strategies to provide emotional support. Students are to observe both verbal and non‐verbal cues in the VR360 experience. Additionally, students will evaluate the situation holistically and propose interventions such as mood monitoring, referral to mental health services and engagement in meaningful activities tailored to the client’s interests and history.

#### 2.4.4. Scenario 4: Challenges With Daily Living Tasks—Grooming and Cooking

In this scenario, students are placed in a 360° view of a client’s apartment within a supported living facility, including a bathroom and a small kitchen. The client, a 76‐year‐old man with moderate Alzheimer’s disease, is observed attempting to complete daily living tasks independently. In the bathroom, he is seen using a disposable razor as a toothbrush, appearing confused but determined. Later, in the kitchen, he begins preparing a meal but forgets to turn off the gas stove after walking away.

The environment includes typical household items, some safety modifications and subtle hazards. Students are expected to identify the cognitive impairments affecting the client’s ability to safely perform grooming and cooking tasks. They will assess the risks associated with these behaviours and evaluate cognitive function, task performance, environmental safety and emotional well‐being. Students are encouraged to propose person‐centred interventions such as task simplification, use of assistive technology, environmental adaptations and caregiver education to support safe and dignified independence.

### 2.5. Procedure

The study began with a pre‐briefing session, during which participants received an orientation on dementia‐related behaviours, the VR360 platform and the CRAT framework. Students then engaged with four immersive VR360 scenarios, each depicting a specific behavioural challenge commonly encountered in dementia care. After each scenario, participants completed the CRAT to self‐assess their clinical reasoning. Following the simulation, participants took part in a facilitated debriefing session and were invited to join optional focus groups to discuss their experiences and perceptions of the learning process.

### 2.6. Quantitative Research Component

To enable a nuanced analysis of learning outcomes, participants were categorised into subgroups based on four key variables: gender, education level, clinical experience and pre‐ and post‐VR360 phases. Gender (male, female) was included to explore potential differences in engagement and cognitive processing styles within immersive virtual environments, consistent with findings from prior research. Education level (undergraduate, postgraduate) was used to assess whether academic maturity influences the development of clinical reasoning skills. Clinical experience (with or without prior dementia care placement) was incorporated into the Cognitive Affective Model of Immersive Learning, which suggests that prior exposure enhances the ability to interpret and respond effectively to complex scenarios [19]. Finally, pre‐ and post‐VR360 phases enabled evaluation of the impact of the VR360 learning module. The combination of these factors resulted in 16 unique subgroups (2 × 2 × 2 × 2), enabling a comprehensive examination of both between‐group and within‐group differences in clinical reasoning across three related domains: content knowledge, procedural knowledge and conceptual reasoning.

Given the interrelated nature of these clinical reasoning domains, MANCOVA was employed to analyse the effects of gender, education level and clinical experience on post‐VR360 scores, whilst controlling for pre‐VR360 scores. This approach allowed for the simultaneous testing of multiple dependent variables and controlled for inflation of Type I error. Pre–post effect sizes were expressed as Cohen’s *d* using the average SD of pre/post scores (paired‐design standardised mean change); partial *η* ^2^ was reported for univariate MANCOVA effects.

### 2.7. Qualitative Research Component

The qualitative strand of this study was designed to explore students’ subjective experiences and reflections following their engagement with the VR360 dementia care scenarios. This component aimed to provide deeper insight into how immersive simulation influences the development of clinical reasoning, complementing the quantitative findings. Semi‐structured focus groups were conducted to further explore participants’ perceptions of the VR360 learning experience, including its realism, emotional impact and perceived influence on their clinical reasoning skills.

A purposive sampling strategy was employed to ensure diversity in gender, education level and prior clinical experience amongst focus group participants. Each group consisted of approximately five students and was facilitated by a trained moderator using a semi‐structured discussion guide. Topics included students’ interpretations of the scenarios, challenges encountered and the perceived value of the CRAT reflection process. All focus groups were audio‐recorded and transcribed verbatim for analysis.

Thematic analysis, following Braun et al.’s [20] six‐phase framework, was used to analyse the qualitative data. This process involves familiarisation with the data, generating initial codes, searching for and reviewing themes, defining and naming themes and producing a final report. NVivo qualitative analysis software was used to support data management and coding. To enhance the trustworthiness of the findings, strategies such as triangulation of data sources (CRAT responses and focus groups), member checking and researcher reflexivity were employed. These measures helped ensure that the analysis accurately reflects participants’ experiences and contributes meaningfully to understanding the impact of immersive learning on clinical reasoning in dementia care.

## 3. Results

Sixty OT students (35 female, 25 male) were recruited from the OT programme at the Hong Kong Polytechnic University through email invitations and module announcements. The sample included both undergraduate (*n* = 40) and pre‐registered postgraduate students (*n* = 20). Participants ranged in age from 19 to 34 years (*M* = 24.6, SD = 4.2). The effectiveness of the VR360 intervention was evaluated using the CRAT, which assessed three domains: content knowledge, procedural knowledge and conceptual reasoning, and the collected CRAT data are depicted in Table 1.

**Table 1:** Participant characteristics and CRAT.

**Characteristic**	**Female (** **n** = 35**)**		**Male (** **n** = 25**)**		**Total (** **N** = 60**)**	

Gender, *n* (%)	35 (58.3%)		25 (41.7%)		60 (100%)	
Undergraduate, *n* (%)	25 (71.4%)		15 (60.0%)		40 (66.7%)	
Postgraduate, *n* (%)	10 (28.6%)		10 (40.0%)		20 (33.3%)	
Age, mean (SD)	24.8 (3.1)		24.3 (3.4)		24.6 (3.2)	

**CRAT scores**	**Pre**	**Post**	**Pre**	**Post**	**Pre**	**Post**

Content knowledge	3.45 ± 0.92	5.45 ± 1.15	3.33 ± 0.92	5.35 ± 1.15	3.40 ± 0.92	5.41 ± 1.15
Procedural knowledge	3.50 ± 0.88	5.60 ± 1.12	3.40 ± 0.88	5.53 ± 1.12	3.46 ± 0.88	5.57 ± 1.12
Conceptual reasoning	3.40 ± 0.87	5.50 ± 0.99	3.37 ± 0.87	5.08 ± 0.99	3.39 ± 0.87	5.32 ± 0.99

Quantitative analysis revealed a marked improvement in students’ clinical reasoning scores across all three domains following the VR360 intervention. The mean pre‐VR360 score for content knowledge was 3.40 (SD = 0.92), which increased to 5.41 (SD = 1.15) post‐VR360. Procedural knowledge showed a similar trend, with scores rising from a pre‐VR360 mean of 3.46 (SD = 0.88) to 5.57 (SD = 1.12) after the VR360 experience. Conceptual reasoning also improved, with the mean score increasing from 3.39 (SD = 0.87) to 5.32 (SD = 0.99). These pre–post changes correspond to large effect sizes: content knowledge (*d* = 1.94), procedural knowledge (*d* = 2.11) and conceptual reasoning (*d* = 2.08). These findings suggest that the VR360 module significantly enhanced students’ ability to identify, interpret and respond to complex behavioural challenges in dementia care.

Assumptions were considered and met based on diagnostic checks. Scatterplots confirmed linearity and homogeneity of regression slopes across subgroups. Box’s *M* test indicated equality of covariance matrices (*p* > 0.001), and Shapiro–Wilk tests suggested approximate multivariate normality. The variance inflation factor (VIF < 2) indicated no multicollinearity amongst dependent variables. These results support the appropriateness of MANCOVA for this dataset. MANCOVA was conducted to examine the effects of gender, education level and prior dementia care experience on post‐VR360 clinical reasoning outcomes—content knowledge, procedural knowledge and conceptual reasoning—whilst controlling for the respective pre‐VR360 scores. The combined dependent variables showed statistically significant multivariate effects for experience (Wilks’ *λ* = 0.517, *F*(3, 51) = 15.88, *p* < 0.001), education level (Wilks’ *λ* = 0.690, *F*(3, 51) = 7.62, *p* < 0.001) and gender (Wilks’ *λ* = 0.802, *F*(3, 51) = 4.21, *p* = 0.010). None of the pre‐VR360 scores were significant covariates, indicating that observed effects were primarily attributable to the VR360 intervention and demographic factors rather than baseline knowledge.

Univariate analyses further clarified the multivariate results, as depicted in Table 2. For content knowledge, a significant effect was observed for dementia care experience (*F*(1, 55) = 17.65, *p* < 0.001, partial *η* ^2^ = 0.24). Gender, education level and pre‐VR360 content scores were not statistically significant predictors in this model (*p*s > 0.05). For procedural knowledge, significant effects were found for both education level (*F*(1, 55) = 14.29, partial *η* ^2^ = 0.21, *p* < 0.001) and dementia care experience (*F*(1, 55) = 28.51, *p* < 0.001, partial *η* ^2^ = 0.34), indicating that postgraduate students and those with prior experience benefited more from the VR360 intervention. Gender and pre‐VR360 procedural knowledge were not significant predictors (*p*s > 0.05).

**Table 2:** MANCOVA results.

Dependent variable	Predictor	*F*(1, 55)	*p*	Partial *η* ^2^
Content knowledge	Experience (yes vs. no)	17.65	< 0.001	0.243
Procedural knowledge	Education (UG vs. PG)	14.29	< 0.001	0.206
Procedural knowledge	Experience (yes vs. no)	28.51	< 0.001	0.341
Conceptual reasoning	Gender (female vs. male)	9.71	0.003	0.150
Conceptual reasoning	Education (UG vs. PG)	10.57	0.002	0.161
Conceptual reasoning	Experience (yes vs. no)	11.65	0.001	0.175

*Note:* df = (1, 55) for all tests. Partial *η* ^2^ computed as *F*/(*F* + 55).

For conceptual reasoning, all three variables—gender (*F*(1, 55) = 9.71, partial *η* ^2^ = 0.15, *p* = 0.003), education level (*F*(1, 55) = 10.57, partial *η* ^2^ = 0.16, *p* = 0.002) and dementia care experience (*F*(1, 55) = 11.65, partial *η* ^2^ = 0.18, *p* = 0.001)—were statistically significant. Effect sizes ranged from 0.15 to 0.34 (partial *η* ^2^), indicating small‐to‐large moderator effects. These results suggest that female students, postgraduates and those with prior dementia care experience demonstrated significantly stronger gains in conceptual reasoning after the VR360 intervention.

Collectively, these findings confirm that the VR360‐based learning module was effective in enhancing clinical reasoning across multiple domains, particularly when paired with prior clinical experience and advanced academic training. Gender differences were also evident in specific cognitive domains, particularly in conceptual reasoning.

Qualitative data gathered from student reflections and focus group discussions further supported these findings. Five key themes, as depicted in Table 3, emerged: (1) increased confidence in managing dementia‐related behaviours, (2) improved ability to identify and interpret behavioural cues, (3) enhanced communication skills with dementia patients, (4) greater empathy and understanding of patient experiences and (5) recognition of the importance of personalised care strategies. Students consistently reported that the immersive nature of the VR360 scenarios helped them better understand the emotional and environmental context of dementia‐related behaviours and allowed them to practise and reflect on their clinical reasoning in a safe, realistic setting.

**Table 3:** Qualitative themes with definitions and representative quotes.

Theme	Definition/focus	Representative quote
Increased confidence	Greater readiness to manage BPSD and apply de‐escalation strategies	‘I feel much more prepared to handle challenging behaviours in dementia care.’
Improved cue recognition	Noticing subtle behavioural/environmental indicators earlier	‘I learned to identify early signs of agitation and intervene before escalation.’
Enhanced communication	More effective trust‐building and person‐centred communication	‘I learned how to build trust with patients who were resistant to care.’
Greater empathy	Deeper understanding of the lived experience of dementia	‘The immersive nature allowed me to see the world from the perspective of a person with dementia.’
Personalised care	Recognition of tailoring interventions to individual preferences	‘It reinforced the value of person‐centred care in my practice.’

### 3.1. Narrative Summary of Student Reflections

The VR360 dementia care learning module had a profound impact on the participating OT students, as evidenced by their reflections. One student expressed increased confidence, stating: ‘After engaging with the VR360 scenarios, I feel much more prepared to handle challenging behaviours in dementia care. The immersive experience allowed me to practise de‐escalation techniques in a safe environment, which boosted my confidence significantly.’ This sentiment was echoed by others who felt more prepared to manage challenging behaviours in dementia care.

Another key theme was cue recognition. A student noted: ‘The VR360 module helped me notice subtle behavioural cues that I might have missed in a real‐world setting. For example, I learned to identify early signs of agitation and intervene before the situation escalated.’ This ability to identify and respond to subtle behavioural cues is crucial in providing effective care for individuals with dementia.

Enhanced communication skills were also a significant outcome of the VR360 module. One student shared: ‘Practising communication strategies in the VR360 scenarios was incredibly valuable. I learned how to build trust with patients who were resistant to care, and I feel more equipped to handle similar situations in my clinical placements.’

This practice in building trust and effectively communicating with patients is essential for successful therapeutic interventions.

The immersive experience fostered a deeper sense of empathy amongst the students. One student reflected: ‘The immersive nature of the VR360 experience allowed me to see the world from the perspective of a person with dementia. This fostered a deeper sense of empathy and understanding, which I believe will make me a more compassionate therapist.’ This increased empathy is likely to translate into more compassionate and patient‐centred care.

Finally, the importance of personalised care was highlighted by the students. One student remarked: ‘The scenarios emphasised the importance of personalised care. I realised that each patient has unique needs and preferences, and it’s crucial to tailor interventions accordingly. This experience reinforced the value of person‐centred care in my practice.’ This recognition of the need for individualised interventions reinforces the core principles of OT.

### 3.2. Triangulation of Quantitative and Qualitative Findings

The integration of quantitative and qualitative findings in this study provides a comprehensive understanding of the impact of the VR360‐based learning module on OT students’ clinical reasoning in dementia care. Quantitative results demonstrated statistically significant improvements across all three domains of clinical reasoning—content knowledge, procedural knowledge and conceptual reasoning—following the VR360 intervention. These gains were particularly pronounced amongst students with prior dementia care experience, postgraduate students and, in the case of conceptual reasoning, female participants. The multivariate analysis confirmed that these demographic factors significantly influenced post‐VR360 outcomes, independent of baseline scores.

Qualitative data from CRAT reflections and focus group discussions reinforced and enriched these findings. Students consistently reported increased confidence in managing dementia‐related behaviours, aligning with the quantitative improvements observed in procedural and conceptual reasoning. The theme of cue recognition, where students described noticing subtle behavioural and environmental indicators, directly supports the observed gains in content and procedural knowledge. Enhanced communication skills, another emergent theme, reflect the students’ improved ability to apply reasoning in real‐time interactions—an essential component of procedural knowledge.

Moreover, the development of greater empathy and a deeper understanding of the lived experience of individuals with dementia aligns with the conceptual reasoning domain, where students must integrate emotional, contextual and theoretical knowledge. The emphasis on personalised care strategies in student narratives further supports the notion that the VR360 module fostered not only technical skill development but also a more holistic, person‐centred approach to clinical reasoning.

Together, these findings suggest that the VR360 intervention was effective not only in improving measurable reasoning skills but also in shaping students’ attitudes, awareness and professional identity. The convergence of statistical outcomes with rich, reflective narratives underscores the value of immersive, experiential learning in preparing OT students for the complexities of dementia care.

## 4. Discussion

This study evaluated the effectiveness of a four‐scenario VR360‐based learning module in enhancing clinical reasoning amongst OT students, particularly in managing behavioural challenges in dementia care. Using a mixed‐methods, quasi‐experimental pre–post design, the study assessed changes in clinical reasoning through both quantitative measures and qualitative insights derived from student reflections and focus group discussions.

### 4.1. Quantitative Findings and Their Implications

Quantitative analysis revealed statistically significant improvements in students’ clinical reasoning across all three domains assessed by the CRAT: content knowledge, procedural knowledge and conceptual reasoning. The magnitude of improvement was large across all three CRAT domains (*d* = 1.94–2.11), consistent with strong learning gains from immersive, context‐rich simulations. These gains suggest that immersive VR360 experiences can effectively support the development of clinical reasoning skills [9]. The structured, emotionally engaging and contextually rich nature of the VR360 module likely contributed to these outcomes by simulating realistic dementia care scenarios that required students to identify, interpret and respond to complex behavioural cues [8].

Multivariate analysis of covariance further revealed that gender, education level and prior dementia care experience significantly influenced post‐VR360 outcomes, independent of pre‐VR360 scores. Moderator effects were small to large (partial *η* ^2^ = 0.15–0.34), with prior dementia care experience showing the largest association, particularly for procedural knowledge. Because the CRAT is a self‐reflection tool and the design is pre–post without a control group, large within‐subject effect sizes should be interpreted as strong proximal learning gains; future controlled trials with objective performance measures can confirm durability and transfer. This indicates that the observed improvements were not merely a function of baseline knowledge but were shaped by the immersive learning experience and individual learner characteristics. Future studies should continue to report these checks to strengthen methodological transparency.

### 4.2. Qualitative Insights: Enriching the Quantitative Narrative

Qualitative data gathered from student reflections and focus group discussions strongly supported and enriched the quantitative findings. Five key themes emerged: (1) increased confidence in managing dementia‐related behaviours, (2) improved ability to identify and interpret behavioural cues, (3) enhanced communication skills, (4) greater empathy and understanding of patient experiences and (5) recognition of the importance of personalised care strategies.

These themes align closely with the CRAT domains. For instance, students’ reports of increased confidence and improved cue recognition directly reflect gains in content and procedural knowledge. This ability to detect and respond to subtle cues is essential in dementia care and reflects the kind of procedural reasoning the CRAT aims to assess. Similarly, the theme of enhanced communication corresponds with improvements in conceptual reasoning, as students practised de‐escalation and trust‐building strategies. This suggests that the immersive environment not only facilitated skill acquisition but also allowed for meaningful reflection and application of therapeutic communication techniques. Additionally, the themes of empathy and personalised care further underscore the value of immersive learning in fostering deeper emotional engagement and patient‐centred thinking. These reflections support the idea that immersive VR can cultivate the affective dimensions of clinical reasoning, which are often difficult to teach through traditional didactic methods [5].

### 4.3. Gender

One of the most notable findings was the significant effect of gender on conceptual reasoning. Female students demonstrated stronger gains in this domain, aligning with prior research suggesting that gender may influence how learners engage with and benefit from immersive virtual environments. For instance, researchers used eye‐tracking data to show that male and female students exhibit different patterns of attention and engagement in VR classrooms, which can affect how they process and retain information [21]. In the context of dementia care, where empathy, emotional regulation and sensitivity to behavioural cues are essential, these gender‐based differences may translate into meaningful variations in clinical reasoning performance.

However, this finding contrasts with earlier work by Ausburn et al. [22], who reported that female students often feel less comfortable and confident in highly technical or visually complex virtual environments. This discomfort could potentially hinder engagement and learning. The discrepancy may be attributed to advancements in VR technology and the increasing emphasis on user‐centred design, which may have made modern VR environments more intuitive and emotionally resonant, particularly in healthcare education. The emotionally immersive nature of the dementia care scenarios may have played to the strengths of female learners, who are often more attuned to emotional and behavioural subtleties.

### 4.4. Care Experience

In contrast to initial expectations, prior dementia care experience emerged as a significant predictor of improved performance across all three CRAT domains. Students with clinical experience demonstrated higher post‐VR360 scores in content knowledge, procedural knowledge and conceptual reasoning. This finding supports theoretical models such as the Cognitive Affective Model of Immersive Learning, which posits that prior knowledge and emotional engagement are key mediators of learning in immersive environments [19]. Students with real‐world exposure to dementia care may have been better equipped to interpret behavioural cues and apply theoretical knowledge in the simulated environment, thereby enhancing their ability to transfer learning from simulation to practice.

This result also aligns with Lin et al. [23], who proposed a two‐path model of VR learning outcomes, suggesting that immersive VR affects learning through both affective (e.g., immersion and enjoyment) and cognitive (e.g., perceived usefulness and active learning) pathways. Students with prior clinical exposure may experience greater cognitive benefits, as they can more readily connect VR content to real‐life clinical reasoning tasks. These students may also experience lower cognitive load and higher confidence, allowing them to engage more deeply with the scenarios and reflect more critically during CRAT assessments.

### 4.5. Education Level

Education level also played a significant role, particularly in procedural and conceptual reasoning. Postgraduate students outperformed undergraduates in these domains, suggesting that advanced academic training may enhance learners’ ability to navigate complex clinical scenarios. This may be due to greater exposure to theoretical frameworks, critical thinking exercises and reflective practice, which are often emphasised at the postgraduate level. These findings underscore the importance of scaffolding VR‐based learning experiences to match students’ academic maturity and cognitive readiness.

Taken together, these findings suggest that immersive VR360 modules, when paired with structured reflection tools like the CRAT, provide preliminary evidence of the potential effectiveness of immersive VR360 learning in OT education. However, the differential effects observed across gender, education level and prior experience highlight the need for more nuanced instructional design. Whilst VR360 has shown promise in education and training, there are challenges to its widespread adoption. These include the need for more robust research designs, such as RCTs, to establish the long‐term effectiveness of VR interventions [9, 24]. Additionally, there is a need for greater integration of pedagogical theories into the design of VR‐based learning activities to ensure that they align with educational goals [9]. Future research should also explore the potential of VR360 to address a wider range of therapeutic needs, such as mental health disorders and neurological rehabilitation. The development of customisable VR environments that can be tailored to individual patient needs is another important area of focus [25].

## 5. Limitations

This study has several limitations that should be acknowledged. First, the absence of a randomised control group is a major limitation. Whilst an RCT would have strengthened internal validity and causal inference, practical and ethical constraints prevented its implementation in this educational setting. Currently, factors such as concurrent coursework, testing effects or natural progression may have contributed to changes in clinical reasoning. Second, the sample size was relatively small and drawn from a single academic institution, which may limit the generalisability of the findings. Third, the use of self‐assessment through the CRAT may introduce bias, as students might overestimate or underestimate their abilities. Additionally, the simulated nature of the VR360 scenarios, whilst immersive, cannot fully replicate the complexity and unpredictability of real‐world clinical environments. Future RCTs with larger and more diverse samples, along with triangulation using objective performance measures to strengthen the evidence base, are needed to confirm the causal attribution of observed improvements to the VR360 intervention and its long‐term impact.

## 6. Conclusion

Overall, the VR360 module yielded large improvements in clinical reasoning and meaningful moderator effects, supporting its adoption as a scalable, person‐centred enhancement to dementia care education. Taken together, the quantitative and qualitative findings present a coherent narrative and provide promising evidence for the effectiveness of immersive VR360 learning in enhancing clinical reasoning amongst OT students. The VR360 module not only improved measurable outcomes across knowledge domains but also fostered emotional engagement, empathy and reflective practice—key components of effective dementia care.

These findings should be interpreted as preliminary and hypothesis‐generating. Rigorous trials, ideally randomised, are needed to establish causal effectiveness and generalisability. Future research should explore how immersive VR can be tailored to support diverse learning needs, including those of neurodiverse students or individuals from underrepresented backgrounds. Longitudinal studies are also needed to assess the retention and transfer of skills acquired through VR‐based training. Additionally, further investigation into gender‐based engagement patterns and the role of digital self‐efficacy could inform the design of more inclusive and effective VR learning environments.

## Funding

This study was funded by the Innovation and Technology Fund for Better Living (ITB/FBL/2004/19/P).

## Ethics Statement

Ethics approval has been granted under the project title ‘VR & AI‐based Mobile Apps in Enhancing Independence of Daily Living in Older Adults and People with Early Dementia’ with reference number HSEARS20191220001.

## Conflicts of Interest

The authors declare no conflicts of interest.

## Data Availability

The data that support the findings of this study are available on request due to privacy and ethical restrictions.
